# Increases survival by apo-sGC activation via post-stroke blood brain barrier stabilisation and anti-inflammation

**DOI:** 10.1186/2050-6511-14-S1-P36

**Published:** 2013-08-29

**Authors:** Friedericke Langhauser, Annegret Hauck, Kim Radermacher, Johannes-Peter Stasch, Christoph Kleinschnitz, Harald HHW Schmidt

**Affiliations:** 1 Neurologische Klinik, University Würzburg, Germany; 2 Dept of Pharmacology, CARIM, Maastricht University, The Netherlands; 3 Bayer HealthCare, Wuppertal, Germany

## Background

Ischemic stroke is the second leading cause of death worldwide. Only one moderately effective therapy exists, albeit with contraindications that exclude 90% of the patients. This medical need contrasts with a high failure rate of more than 1,000 pre-clinical drug candidates for stroke therapies. Thus, there is a need for translatable mechanisms of neuroprotection and more rigid thresholds of relevance in pre-clinical stroke models. In many settings of ischemia, vasodilation to increase perfusion is a suitable approach; yet in stroke it bears the risk of systemic hypotension, shunting of blood from the ischemic to healthy areas, increased infarcts and eventually reduced survival. One potentially innovative and mechanism-based approach are sGC activators, as they represent disease-specific vasodilators that are potentiated under conditions of oxidative stress and have microvascular selectivity e.g. to unload the acutely failing heart.

## Results

Here we show that in the experimental stroke model of transient middle cerebral artery occlusion (tMCAO) in the mouse, substantial oxidative stress leads to 100% mortality after one week. However, post-stroke treatment with the sGC activator, BAY 60-2770, increased cerebral blood flow, prevented the breakdown of the blood brain barrier together with less apoptosis and smaller infarct volumes. This was associated with an increase in the biomarkers of sGC-cGMP signaling, P-VASP. As expected this increase in cGMP was not affecting oxidative stress directly but surprisingly exerted a potent anti-inflammatory response on microglia activation and neutrophil infiltration and inhibited neuronal apoptosis. Systemic blood pressure, a potential confounder, was not affected. Besides these surrogate parameters, importantly survival was dramatically enhanced and despite the severity of the model after 7 days 50% of the animals survived. When analysisng specific sources of ROS that may lead to sGC oxidation and heme loss, we observed that in NADPH oxidase 4 (NOX4) KO mice, P-VASP levels were dramatically increased versus wild-type mice.

## Conclusion

Thus in stroke NOX4-derived oxidative stress may contribute to sGC oxidation and haem loss and subsequent apo-sGC activation represents a disease-specific therapeutic intervention leading to blood- brain barrier stabilisation, a substantial anti-inflammatory component and increased survival.

